# The Acute and Chronic Cognitive and Cerebral Blood Flow Effects of a *Sideritis scardica* (Greek Mountain Tea) Extract: A Double Blind, Randomized, Placebo Controlled, Parallel Groups Study in Healthy Humans

**DOI:** 10.3390/nu10080955

**Published:** 2018-07-24

**Authors:** Emma L. Wightman, Philippa A. Jackson, Julie Khan, Joanne Forster, Felix Heiner, Bjoern Feistel, Cynthia G. Suarez, Ivo Pischel, David O. Kennedy

**Affiliations:** 1 Brain Performance and Nutrition Research Centre (BPNRC), Northumbria University, Newcastle Upon Tyne NE1 8ST, UK; philippa.jackson@northumbria.ac.uk (P.A.J.); julie.khan@northumbria.ac.uk (J.K.); jo.forster@northumbria.ac.uk (J.F.); david.kennedy@northumbria.ac.uk (D.O.K.); 2 Finzelberg GmbH & Co. KG, Martin-Bauer-Group, 56626 Andernach, Germany; Felix.Heiner@finzelberg.de (F.H.); Bjoern.Feistel@finzelberg.de (B.F.); CynthiaGisela.SuarezRizzo@finzelberg.de (C.G.S.); 3 Dr. Ivo Pischel Consulting, Lacher Weg 1, 53547 Rossbach, Germany; info@drpischel.com

**Keywords:** *Sideritis scardica*, Greek mountain tea, *Ginkgo biloba*, cognition, mood, cerebral blood flow

## Abstract

Background: The presence of polyphenols such as hydroxy-cinnamic acids and flavonoids in *Sideritis scardica* (Greek mountain tea) are likely responsible for the cognitive and mood effects of its consumption and this could be underpinned by the ability of such polyphenols to prevent monoamine neurotransmitter reuptake and to increase cerebral blood flow (CBF). Objective: The current study extends the small amount of *Sideritis scardica* literature in humans by assessing both cognitive and mood outcomes in a sample of older adults, as well as blood pressure (BP) and CBF, in a subsample, utilizing near-infrared spectroscopy (NIRS). Design: This randomized, double-blind, placebo-controlled, parallel groups trial randomized *N* = 155, 50–70-year-old male and female participants who were assessed for the cognitive (*N* = 140), mood (*N* = 142), BP (*N* = 133) and CBF (*N* = 57) effects of two doses of Greek mountain tea (475 and 950 mg) as well as an active control of 240 mg *Ginkgo biloba*, and a placebo control, following acute consumption (Day 1) and following a month-long consumption period (Day 28). Results: Relative to the placebo control, 950 mg Greek mountain tea evinced significantly fewer false alarms on the Rapid Visual Information Processing (RVIP) task on Day 28 and significantly reduced state anxiety following 28 days consumption (relative also to the active, Ginkgo control). This higher dose of Greek mountain tea also attenuated a reduction in accuracy on the picture recognition task, on Day 1 and Day 28, relative to Ginkgo and both doses of Greek mountain tea trended towards significantly faster speed of attention on both days, relative to Ginkgo. Both doses of Greek mountain tea, relative to placebo, increased oxygenated haemoglobin (HbO) and oxygen saturation (Ox%) in the prefrontal cortex during completion of cognitively demanding tasks on Day 1. The higher dose also evinced greater levels of total (THb) and deoxygenated (Hb) haemoglobin on Day 1 but no additional effects were seen on CBF on Day 28 following either dose of Greek mountain tea. *Ginkgo biloba* led to lower levels of Ox% and higher levels of Hb on Day 1 and lower levels of both HbO and THb on Day 28. Conclusions: The significantly improved cognitive performance following Greek mountain tea on Day 1 could be due to significant modulation of the CBF response. However, these improvements on Day 28 are more likely to be due to the reductions in state anxiety and, taken together, suggests that the former mechanism is more likely to facilitate acute cognitive effects and the latter more likely to underpin more prolonged cognitive improvements.

## 1. Introduction

Shepherd’s Tea, Olympus tea or, interchangeably referred to herein as, Greek mountain tea is a popular naturally un-caffeinated Eastern European beverage derived from *Sideritis scardica* (ironwort), a hardy perennial which is renowned for flourishing at high altitude. As with many traditional products ascribed “healing” properties—in this case respiratory, digestion and inflammation are chief amongst them [1]—one finds that modern evidence supports the curative myths of Greek mountain tea. In a recent review, Todorova and Trendafilova [2] reported gastro-protective and anti-inflammatory effects in vitro alongside a correlation between antioxidant activity and the phenolic content of the tea extract.

The primary active constituents of the caffeine-free Greek mountain tea appear to be phenolic acids, principally ferulic acid and chlorogenic acid and the flavone compound apigenin [3], and it is likely the latter which underpins the small number of published psychophysiological effects of Greek mountain tea in humans. *Sideritis scardica* is a triple monoamine (serotonin, noradrenaline and dopamine) reuptake inhibitor in vitro [4] with apigenin individually demonstrating a capacity to interact with monoamine transporters [5] as well as being a ligand for the benzodiazepine receptor. This latter effect results in anxiolytic effects in mice during the stressful elevated plus maze task and may underlie the small number of mood improving effects in humans published recently.

Behrendt et al. [6], for example, observed cognitive improvements in a sample of 64 healthy males and females (25–60 years) following six-week supplementation of 330 mg/daily *Sideritis scardica*, including during stressful scenarios such as the presence of noise and incongruent stimuli. This cognitive improvement is extended to those experiencing mild cognitive impairment; here Dimpfel et al. [7] reported trending improvements on tasks of concentration, arithmetic calculation and memory in 32 males and females, aged 50–80 years, following four-week supplementation of 380 mg/daily *Sideritis scardica*. However, whilst these trials showed promising effects, both studies co-supplemented Greek mountain tea with B vitamins and the latter also incorporated 120 mg *Bacopa monnieri*. This makes attributions to *Sideritis* alone complicated as both B vitamins and *Bacopa* have the capacity to influence cognition and mood, including in the elderly [8,9]. Hence, the current study extends this with a more robust trial investigating *Sideritis scardica* alone.

Chlorogenic and ferulic acid also have a small amount of literature which supports potential cognitive effects in humans. The former is an abundant phenolic acid in the coffee berry and, in turn, caffeine supplements/coffee, making isolating its individual contribution to cognition difficult. However, Camfield et al. [10] isolated chlorogenic acid and supplemented a cohort of 39 elderly (53–79 years) adults with a low (224 mg) or high (521 mg) decaffeinated beverage, finding increased alertness and emotional processing bias and a tendency towards decreased headache and mental fatigue with the higher dose. However, in comparison, the effects of the caffeinated chlorogenic coffee drinks were far greater, suggesting that the presence of caffeine and/or other coffee components (e.g., other phenolics) are required to exert more robust effects on cognition.

In terms of mechanisms, both ferulic [11] and chlorogenic [12] acid have demonstrated the capacity to interact with the vasodilatory mediator nitric oxide (NO) and this has resulted in peripheral blood flow effects in animal models [13] and humans; here, lowered blood pressure was observed in healthy adults following 400 mg chlorogenic acid [14] and increased oxygen saturation was seen in the prefrontal cortex following a chlorogenic acid-rich coffee berry extract [15]. Whilst no further data exist with these phenolic compounds with regards NO, vasodilation and subsequent effects, more advanced research with other polyphenols provides a sound hypothetical rationale for anticipating effects here. For example, both cocoa flavanol polyphenols and the individual stilbene polyphenol resveratrol have a growing body of evidence which supports their capacity to promote vasodilation of the vasculature [16,17,18]. This, in turn, increases blood flow, including to the brain [19,20,21], which is likely related to their ability to increase NO [22,23]. The ensuing increase in blood borne neural fuels, oxygen and glucose, to the brain following increased blood flow here has proved an elusive mechanism to tie directly to cognitive improvements. Nevertheless, cognitive improvements have been observed, with cocoa polyphenols in particular [24,25,26].

Taken together, the active components of Greek mountain tea are predominantly phenolic acids and evidence from these, and related, phenolic compounds suggest that a Greek mountain tea extract may also evince cognitive and mood effects. The mechanisms underpinning these effects are likely to be NO modulation of CBF and interaction with monoamine neurotransmitter systems respectively. The current study aimed to test this in a cohort of healthy, older adults over an acute and chronic time-frame. Because the cognitive and CBF effects of Greek mountain tea are presently unknown, the current study utilized an active control (*Ginkgo biloba* extract, Ph. Eur. quality) as well as a placebo control. Ginkgo has established sensitivity to key aspects of this methodology [27] and, in addition, the purported mechanisms underpinning the psychoactive effects of Ginkgo align with Greek mountain tea, namely CBF modulation [28,29]; thus, taken together, it lends itself well as an active comparator here.

## 2. Methods

### 2.1. Study Design and Participants

**Figure 1:**
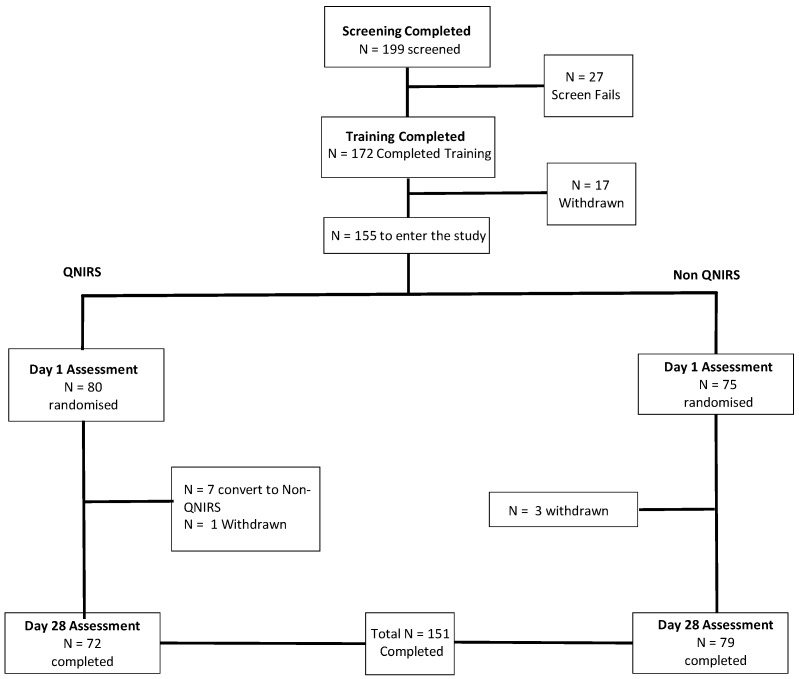
Participant disposition through the trial. Figure depicts the disposition of participants throughout the study, which culminating in *N* = 151 of the 155 who were randomised. A single participant in the placebo group withdrew due to adverse event (vomiting, which a follow-up reported had been remedied and the participant was in good health) and the remaining three withdrawals left due to other time commitments or lack of continued interest in the study. QNIRS, Quantitative Near-Infrared Spectroscopy.

The number of participants randomized into this double-blind, placebo-controlled, parallel groups trial was *N* = 155 (mean age 60.3 years, 95 female, 60 male, 143 right handed, 12 left handed, and an average of 16.3 years in education). Here, the acute and chronic effects of three doses of active treatment, plus placebo, on cognition were assessed pre-dose and at 1.5 and 4.5 h post-dose on Day 1 and Day 28. A sub-set of participants also provided CBF data on both days (see Figure 1). For disposition of participants through the trial, exclusion criteria were: BMI < 18 or >35 kg/m^2^; high blood pressure (defined as systolic >159 mmHg or diastolic >99 mmHg); smoking; food allergies or insensitivities; pregnancy; breast feeding; currently taking any medication (use of contraceptives/hormone replacements, if stable for minimum of three months, was not excluded) or dietary supplements which would contraindicate with the study; sleep disturbances and/or taking sleep aid medication; history of neurological, vascular or psychiatric illness; current diagnosis of anxiety or depression; migraines; recent history (within 12 months) of alcohol/substance abuse; disorder of the blood; heart disorder/history of vascular illness; respiratory disorder requiring regular medication; Type I or II diabetes; renal disease; hepatic disease; severe disease of the gastrointestinal tract, and any health condition that would prevent the fulfilment of the study requirements.

The withdrawal of four participants (three due to time commitments and one due to sickness (the latter participant was in the placebo control condition)) resulted in a final dataset of *N* = 151. *N* = 4 were then removed pre-analysis due to protocol violations associated with prohibited medication use. A further *N* = 7 were then identified for removal due to non-adherence to the dosing regimen during the final week prior to the chronic lab visit. Thus, a final dataset of *N* = 140 was arrived at for the cognitive analyses (mean age 60.4 years, 84 females, 46 males, 130 right handed, 10 left handed, and average 16.3 years in education). Data catchment errors resulted in a sample of *N* = 142 for the mood (State Trait Anxiety Inventory (STAI)) analysis (mean age 60.3 years, 85 females, 57 males, 133 right handed, 9 left handed, and average 16.3 years in education). Data catchment errors also resulted in a reduced BP sample of *N* = 133 (mean age 60.3 years, 81 females, 52 males, 124 right handed, 9 left handed, and average 16.3 years in education). For the NIRS analysis, data collection was attempted for a subset of *N* = 70 participants. The blind data review highlighted the removal of *N* = 1 dataset pre-analysis due to protocol deviations concerning medication use. This was further reduced by removal of *N* = 8 partial datasets due to data catchment errors. *N* = 4 were then removed due to outlying CBF levels. This resulted in a final sample for analysis of *N* = 57 (mean age 60.1 years, 31 female and 26 male, 51 right handed, 6 left handed, and average number of years spent in education 16.9 years).

This study was conducted according to the guidelines laid down in the Declaration of Helsinki and all procedures were approved by the department of Psychology (Northumbria University) ethics committee (code: SUB010_Khan_141116). Written informed consent was obtained from all participants.

### 2.2. Treatments

Participants were randomized via Latin square into one of four treatment conditions which all involved the consumption of two capsules daily, creating:

1. Placebo (coloured maltodextrin Ph. Eur.)
2. 475 mg *Sideritis scardica* extract
3. 950 mg *Sideritis scardica* extract
4. 240 mg *Ginkgo biloba* extract Ph. Eur. (representing an active control)

The *Sideritis scardica* extract was manufactured under validated Good Manufacturing Practice (GMP) conditions and extensively characterized by various chromatographical and spectrophotometrical methodologies. This revealed a total phenolic content of 6.25% of the 20% EtOH (*v*/*v*) *Sideritis* extract with the major phenolic classes represented by flavonoids (1.2%), such as scutellarin, apigenin, luteolin, etc. and their glycosides, phenolic acids, e.g., caffeoyl quinic acids (0.5%), and acteoside (0.4%).

Participants consumed their first (acute: Day 1) and last (chronic: Day 28 ± 3 days) treatments in the lab (consuming both capsules at approximately 10:00 a.m.). In the intervening period, participants self-supplemented by taking one capsule in the morning (advised “with breakfast”) and one capsule in the evening (advised “with evening meal”) from identical white pots that contained more capsules (also identically white) than the dosing regimen required. These pots were prepared and labelled for randomization by a third party who had no further involvement with the study. The surplus number of returned capsules varied between participants and a count of the returned capsules served as the primary compliance measure. A second compliance measure included a capsule diary which required participants to note the time of each capsule consumption.

### 2.3. Cognitive and Mood and Blood Pressure (BP) Assessment

All cognitive function tests were delivered using the Computerised Mental Performance Assessment System (COMPASS, BPNRC, Newcastle Upon Tyne, UK). This testing system delivers a bespoke collection of tasks, with fully randomised parallel versions of each task delivered at each assessment for each individual. The battery has been in use within the BPNRC for over 10 years and is now commercially available for other research organisations (www.cognitivetesting.co.uk) and is currently in use within a number of UK, US, New Zealand, and Australian Universities, companies and research organisations.

**Figure 2:**
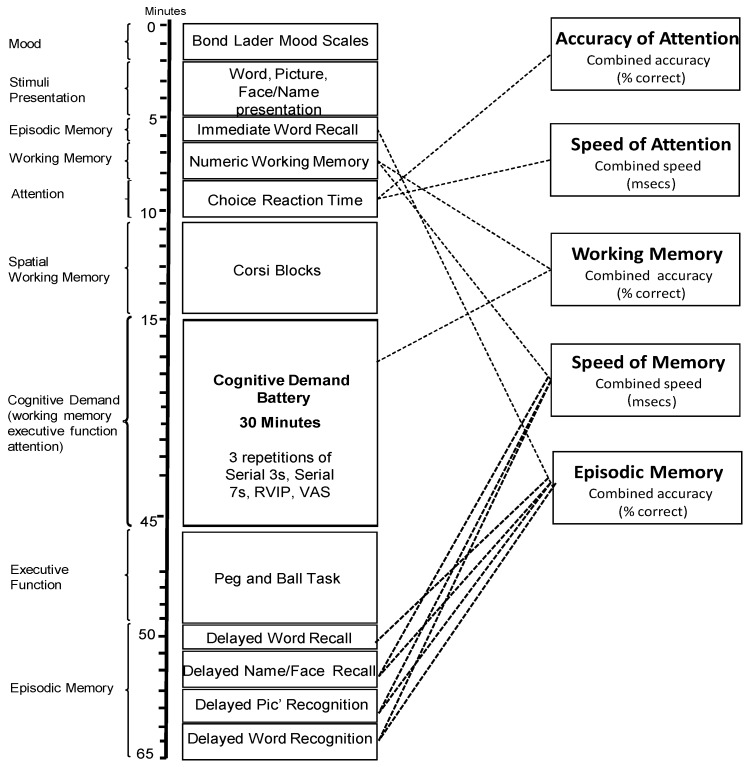
Cognitive task battery. Figure depicts the order of cognitive task completion during the pre-dose and two post-dose cognitive task battery completions. The left of the figure shows the approximate task timings and predominant cognitive domain of each task. The right of the diagram demonstrates how global cognitive domains can be derived from outcome measures of individual tasks. RVIP, Rapid Visual Information Processing; VAS, Bond Lader Visual Analog Scales.

In the face of little evidence of domain-specific cognitive effects of *Sideritis scardica*, this paradigm is somewhat exploratory with the cognitive tasks used herein assessing multiple facets of cognition. Figure 2 outlines the individual tasks used (with these task procedures described elsewhere [30]), the order in which they were presented and the approximate timings as well as the primary cognitive domain of each task (left-hand-side of the diagram) and the collapsible global outcome measures (right-hand-side).

To assess mood, the current study used both the Bond Lader [31] mood scales (completed at the beginning of each task battery repetition) and the State Trait Anxiety Inventory (STAI) [32]. Both state and trait anxiety were measured during the screening/training visit to provide a baseline measure of mood. Subsequently, during each testing session, only state was assessed as trait mood should be stable across this relatively short period.

Sitting blood pressure and heart rate readings were collected using a Boso Medicus Prestige (BOSCH + SOHN GmbH u. Co. KG, Jungingen, Germany) blood pressure monitor with the subject’s arm supported at the level of the heart and with their feet flat on the floor. Readings were taken following completion of the baseline tasks and again following completion of the post-dose tasks.

## 3. Near-Infrared Spectroscopy (NIRS) Analysis

Haemodynamic response was monitored using a frequency domain “quantitative” NIRS system (OxiplexTS Frequency-Domain Near-Infrared Tissue Oximeter; ISS, Inc., Champaign, IL, USA). NIRS has proven efficacy in studying haemodynamic activation in humans during activated brain function [33] and this system gives absolute measurements of absorption of near-infrared light emitted at two distinct wavelengths which allows for the quantitation of oxygenated haemoglobin (HbO_2_) and deoxygenated haemoglobin (HHb). These values are then used to determine total haemoglobin (tHb; HbO_2_ + HHb) and oxygen saturation (Ox%; HbO_2_/tHb × 100%). This system is ideal for quantifying acute changes in haemodynamic response over an extended period (i.e., with intermittent testing throughout one visit) and in a chronic context (comparing CBF between Day 1 and Day 28).

Light was emitted at 691 and 830 nm by optical fibres glued in pairs to four prisms (eight fibres in total) that were separated from the collector bundle, also glued to a prism, by 2.0, 2.5, 3.0 or 3.5 cm. Each of the emitter and collector bundle prisms were embedded into a flexible polyurethane resin to form a sensor with the overall dimensions of 7.6 cm × 2.5 cm × 0.3 cm. Identical sensors were attached to either side of the forehead of participants with medical tape and secured in place with a self-adhering bandage. The sensors were positioned so that the bottom edge was level with the top of the participants’ eyebrows and the middle edge touching at the midline of the forehead. Data were collected at a rate of 5 Hz.

### 3.1. Procedure

Participants attended the laboratory at Northumbria University, UK, between February and August 2017 on three separate occasions. An initial training/screening visit was conducted 1–14 days before their first active, acute, visit to the lab. Twenty-eight days later (±3 days), the participant returned for their second active, chronic, visit to the lab. If more than 14 days elapsed between training and the first active study session, then participants were invited to return for a “refresher” training visit before commencing the study.

The screening/training visit to the laboratory comprised: briefing on requirements of the study, obtaining of informed consent, health screening, completion of the Caffeine Consumption Questionnaire (CCQ) and STAI, training on the cognitive and mood measures and collection of demographic data.

For both active, laboratory-based testing sessions, participants presented at 8:30 a.m. having consumed a standardised breakfast of cereal and/or toast at home no later than 7:30 a.m. and confirmed that they had refrained from alcohol for 24 h and caffeine for 18 h. Participants first completed the STAI (state subscale) and the computerised cognitive assessment (as per Figure 2) followed by measurements of BP and heart rate. As mentioned above, a sub-set of participants provided additional CBF measures at points during the testing day and here a maximum of two participants per day provided a baseline measure by wearing the NIRS headband for 5 min. Following this, all participants consumed their treatment for the day (~10:00 a.m.). Two further cognitive assessments (followed immediately by BP/HR) commenced at 90 (~11:30 a.m.) and 310 (~2:30 p.m.) min post-dose. In between the second and third cognitive assessments of the day, the NIRS sample provided a post-dose measure of CBF. This comprised a 5 min post-dose baseline followed by 30 min of cognitive tasks (cognitive demand battery (30) × 3). Participants were provided with a standardised lunch between the second and third post-dose cognitive assessments (for the NIRS participants, one was accommodated before lunch and one after) at ~1:20 p.m.

**Figure 3:**
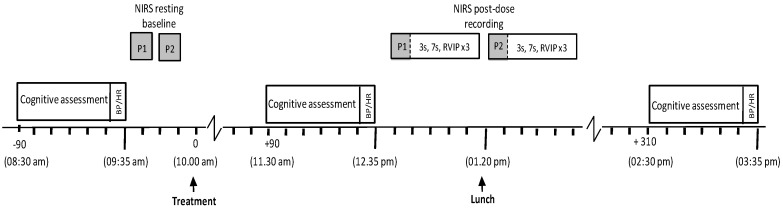
Testing session timeline for both active lab visits. Figure details the itinerary of the two acute lab visits with participants completing the pre-dose cognitive task battery (detailed in Figure 2), and blood pressure (BP) reading, at ~8:30 a.m. Each day, two participants could then be scheduled to provide pre-dose cerebral blood flow (CBF) readings before all participants consumed treatment at ~10:00 a.m. Ninety minutes later, the first post-dose cognitive task battery, and BP, was completed, after which the aforementioned sub-sample of participants provided post-dose CBF data (5 min rest followed by three completions of the 10 min cognitive demand battery) before/after lunch. At 310 min post-dose, all participants then completed the second post-dose task battery and BP and the day was completed.

Lunch comprised a cheese sandwich (Hovis soft white bread × 2 slices: 186 kcal, 1.4 g fat, 2.8 g sugar, 7 g protein; Sainsbury’s British Medium Grated Cheddar Cheese 30 g: 127 kcal, 10.5 g fat, <0.5 g sugar, 7.6 g protein; Lurpack slightly salted spread ~10 g: 72 kcal, 8 g fat, <0.1 g sugar, <0.1 g protein), one packet of ready salted flavour crisps (Walkers 25 g bag: 132 kcal, 8 g fat, 0.1 g sugar, 1.5 g protein) and one pot of custard (Ambrosia 125 g pot (due to an ordering error, some participants consumed the light version (values in italics) for both visits): 124/113 kcal, 3.5/2.3 g fat, 14.3/13.8 g sugar, 3.6/3.6 g protein). This lunch was optional (as long as non/consumption of components was the same for both visits) to avoid the potentially more disruptive effects of eating items which were unpalatable to participants. However, with only a few exceptions of individual items not consumed, all participants ate lunch each day. See Figure 3 for full testing day procedure.

### 3.2. Statistics

A G*Power [34] calculation determined that, to achieve a large effect size (80% power), with four groups, utilizing the following analysis plan, would require a sample size of *N* = 144.

All of the below analyses were first investigated for baseline differences between treatment groups and, unless reported, no baseline differences were detected. The confidence intervals for all analyses are set at 95%. The analyses for the primary outcome measures (cognition and mood) and secondary outcome measures (BP and CBF) were as follows.

#### 3.2.1. Cognitive, Mood (Bond-Lader) and Blood Pressure Assessment

Two ANOVAs were conducted to determine if acute effects of treatment had taken place within Day 1 and/or Day 28 as well as whether any effects seen on Day 28 were due to acute effects of consuming treatment on that day alone or the result of an accumulative effect of 28 days of treatment consumption.

For the initial “acute” ANOVA, cognitive and BP data were converted to change from baseline with respect to the pre-treatment scores on the first day of treatment (Day 1) and analysed via a repeated measures ANOVA (treatment (475 mg Greek mountain tea/950 mg Greek mountain tea/240 mg Ginkgo biloba/placebo) × repetition (×5 (representing post-dose repetitions 1 and 2 on Day 1 and pre-dose and post-dose repetitions 1 and 2 on Day 28)).

To ascertain whether any Day 28 effects were due to the chronic dosing regimen, pre-dose data on Day 28 were converted to change from Day 1 pre-dose baseline and analysed via one-way ANOVA to compare performance between treatments. A significant effect here, before treatment had been consumed on Day 28, would indicate a pure chronic effect of treatment due to 28 days of consumption. If acute effects were also seen on the above ANOVA on Day 28, i.e., an effect after treatment had then been consumed, then these effects can be attributed to this chronic effect.

If any main and/or interaction effects which included the “treatment” factor were observed on the above ANOVAs, then Bonferroni corrected post-hoc student t tests were conducted to assess where these differences occurred. This analysis plan has proven sensitivity in detecting the acute and chronic effects of the polyphenol resveratrol in healthy, human participants previously [20].

#### 3.2.2. Mood (State Trait Anxiety Inventory)

The trait subscale was used to investigate potential baseline differences between the four treatment groups via a Tukey corrected univariate ANOVA. The Day 1 state anxiety score was subtracted from the Day 28 score and this change value was compared between the four treatments via a Tukey corrected univariate ANOVA.

#### 3.2.3. Near-Infrared Spectroscopy (NIRS) Assessment

Data were first averaged across the two hemispheres and converted to change from baseline (this being an average of the 5 min recording taken after the pre-dose cognitive task battery completion and just before treatment consumption) and then averaged into 2 min (or 2.5 min when the period (i.e., the RVIP task and rest periods) was 5 min long) epochs for analysis. Analysis was via ANOVA utilizing treatment (475 mg Greek mountain tea/950 mg Greek mountain tea/240 mg *Ginkgo biloba*/placebo) × epoch (equalling 14 time-points following the above data condensing) × day (Day 1 and Day 28) as factors. If significant main or interaction effects were seen here, then uncorrected post-hoc planned comparisons were conducted comparing the three active treatments (including Ginkgo, the active control) to the placebo control at each epoch, resulting in 42 separate planned comparisons.

## 4. Results

### 4.1. Compliance and Treatment Guess

The treatment consumption period lasted 28 ± 3 days (ranging 25–31 days) with mean compliance, as determined by a returned capsule count, 102%; ranging 76–114%. *N* = 135 participants completed a treatment guess response at the end of Day 28 with *N* = 83 indicating that they believed they were taking placebo (*N* = 64 of whom were correct) and *N* = 52 (*N* = 19 of whom were correct) taking one of the three active treatments. A Chi-square analysis revealed that there was no significant difference between the ability to correctly detect the active treatments and not χ (1) = 0.08, *p* = 0.779.

### 4.2. Cognitive and Mood Assessment

#### 4.2.1. Cognitive Performance

Due to the number of statistical analyses performed, only those which revealed significant main or interaction effects including treatment are reported below. However, all results are reported in Supplementary Materials (see Table S1).

#### 4.2.2. Picture Recognition Accuracy

A significant interaction between repetition × treatment F (12, 544) = 1.84, *p* = 0.040 was seen where mean performance by those consuming 950 mg Greek mountain tea was significantly higher than those consuming Ginkgo. Post-hoc t tests revealed that these differences lay between Ginkgo and 950 mg Greek mountain tea at repetitions 1 (*p* = 0.023, *d* = 0.68), 2 (*p* = 0.026, *d* = 0.61) (i.e., both post dose repetitions during Day 1) and 5 (*p* = 0.005, *d* = 0.79) (i.e., the final post-dose repetition during Day 28).

**Figure 4:**
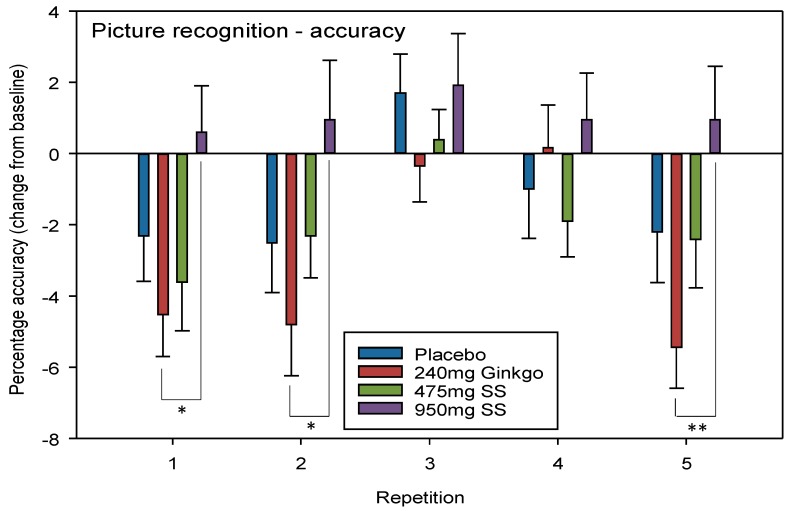
Picture recognition accuracy. Mean (±SEM) change-from-baseline scores on picture recognition accuracy (percentage correct) on Day 1 (repetition 1 = post-dose repetition 1 on Day 1, repetition 2 = post-dose repetition 2 on Day 1) and following 28 days of supplementation with 475 mg *Sideritis scardica* (SS), 950 mg SS, active control (240 mg *Ginkgo biloba*) or placebo control (repetition 3 = pre-dose baseline on Day 28, repetition 4 = post-dose repetition 1 on Day 28 and repetition 5 = post-dose repetition 2 on Day 28), * *p* < 0.05, ** *p* < 0.01.

No significant effect of day F (1, 136) = 2.6, *p* = 0.109 or a treatment × day interaction F (3, 136) = 0.941, *p* = 0.423 was seen when comparing pre-dose data across treatments on Day 28. See Figure 4 for picture recognition accuracy treatment × repetition interaction effects.

#### 4.2.3. Rapid Visual Information Processing (RVIP) False Alarms (FA)

A significant interaction between repetition × treatment F (9, 409) = 2.08, *p* = 0.030 was observed. Post-hoc *t*-tests revealed that these differences lay between placebo and 950 mg Greek mountain tea at repetition 4 only (*p* = 0.017, *d* = 0.66).

**Figure 5:**
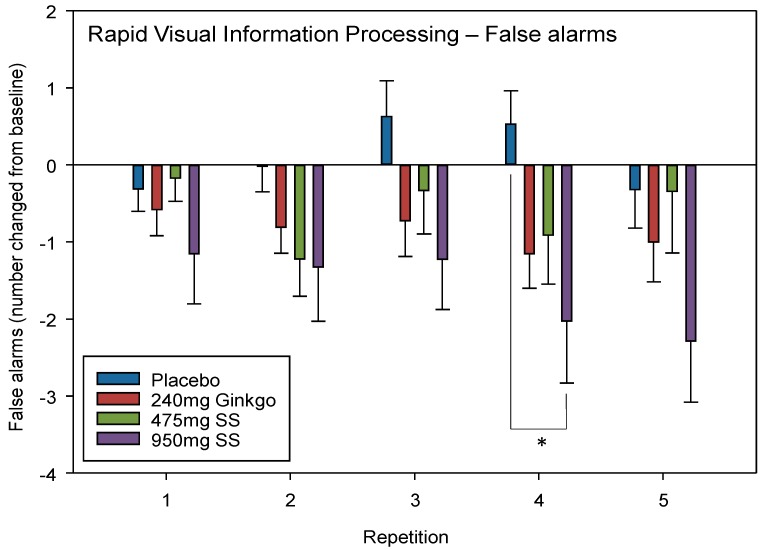
Rapid Visual Information Processing (RVIP) False alarms. Mean (±SEM) change-from-baseline false alarms on the RVIP task on Day 1 (repetition 1 = post-dose repetition 1 on Day 1, repetition 2 = post-dose repetition 2 on Day 1) and following 28 days of supplementation with 475 mg *Sideritis scardica* (SS), 950 mg SS, active control (240 mg *Ginkgo biloba*) or placebo control (repetition 3 = pre-dose baseline on Day 28, repetition 4 = post-dose repetition 1 on Day 28 and repetition 5 = post-dose repetition 2 on Day 28). * *p* < 0.05.

No significant effect of day F (1, 134) = 2.41, *p* = 0.123 or a treatment × day interaction F (3, 134) = 2.16, *p* = 0.096 was seen when comparing pre-dose data across treatments on Day 28. See Figure 5 for RVIP FA treatment × repetition interaction effects.

#### 4.2.4. Speed of Attention

Speed of attention is 1/5 global cognitive measures (see Figure 2) which can be derived by collapsing individual task outcome measures. Here, speed of attention comprises averaged speed (ms) from numeric working memory, choice reaction time and rapid visual information processing tasks.

A trend towards significance was observed for repetition × treatment F (10.4, 469) = 1.65, *p* = 0.088. Reference to post-hoc comparisons reveals that the effects lie between Ginkgo and 475 mg Greek mountain tea at repetition 1 (*p* = 0.008, *d* = 0.66) as well as between Ginkgo and 950 mg Greek mountain tea at repetition 1 (*p* = 0.050, *d* = 0.54). The same pattern of effects was observed at repetitions 2 (Ginkgo V 475 mg Greek mountain tea, *p* = 0.086, *d* = 0.51; Ginkgo V 950 mg Greek mountain tea, *p* = 0.015, *d* = 0.63) and 5 (Ginkgo V 475 mg Greek mountain tea, *p* = 0.021, *d* = 0.59; and Ginkgo V 950 mg Greek mountain tea, *p* = 0.005, *d* = 0.74).

**Figure 6:**
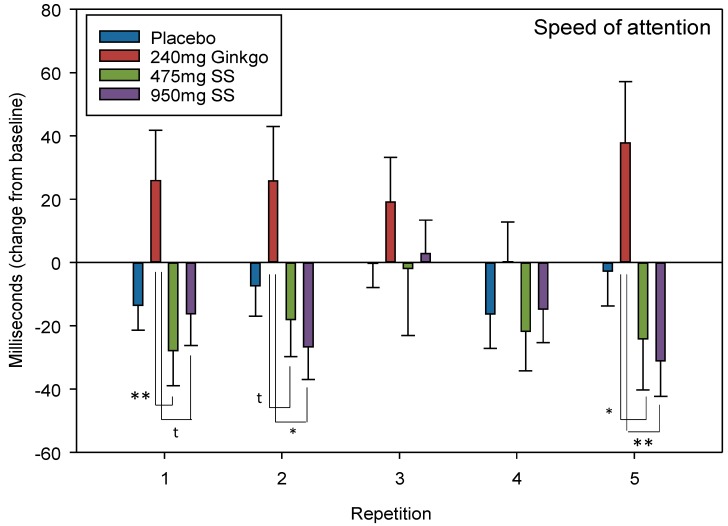
Speed of attention. Mean (± EM) change-from-baseline performance on “speed of attention” (derived from collapsing reaction time data from “numeric working memory”, “choice reaction time” and the “Rapid Visual Information Processing” task) on Day 1 (repetition 1 = post-dose repetition 1 on Day 1, repetition 2 = post-dose repetition 2 on Day 1) and following 28 days of supplementation with 475 mg *Sideritis scardica* (SS), 950 mg SS, active control (240 mg *Ginkgo biloba*) or placebo control (repetition 3 = pre-dose baseline on Day 28, repetition 4 = post-dose repetition 1 on Day 28 and repetition 5 = post-dose repetition 2 on Day 28). * *p* < 0.05, ** *p* < 0.01 and *t* = trend.

A significant effect of day F (1, 136) = 31.1, *p* ≤ 0.001 but no interaction between day × treatment F (3, 136) = 0.509, *p* = 0.677 was observed when comparing pre-dose data across treatments on Day 28. See Figure 6 for speed of attention treatment × repetition interaction effects and Table 1 for summary of the ANOVA and post-hoc (including effect sizes) results for the above significant cognitive outcomes.

#### 4.2.5. Mood–Bond–Lader Visual Analogue Scales (VAS)

No significant main or interaction effects involving treatment were observed. The data for these scales are provided in Table 1.

#### 4.2.6. Mood—State-Trait Anxiety Inventory (STAI)

A Tukey corrected univariate ANOVA was conducted to compare the trait score among the four treatment groups on Day 1 to ascertain if any intrinsic differences existed. No significant difference was found F (3, 138) = 1.44, *p* = 0.234

**Figure 7:**
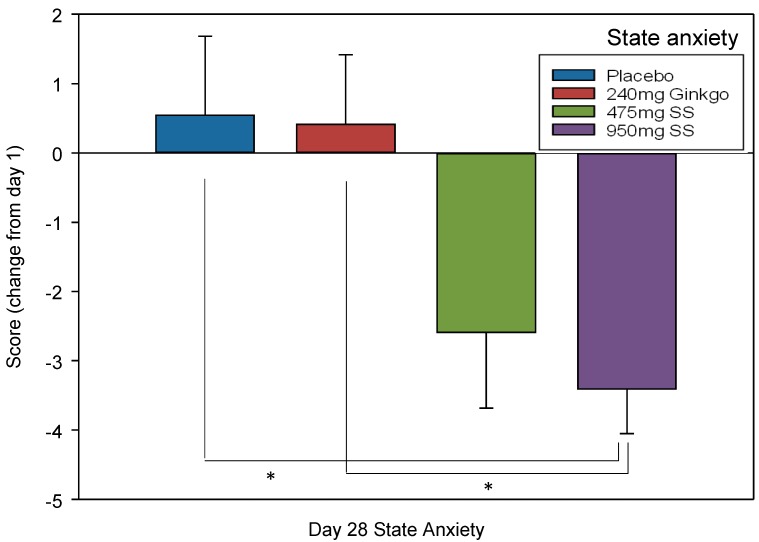
State anxiety change. Mean (±SEM) subjective state anxiety ratings (changed from Day 1 rating) of participants following 28 days of supplementation with 475 mg *Sideritis scardica* (SS), 950 mg SS, active control (240 mg *Ginkgo biloba*) or placebo control. * *p* < 0.05.

To investigate whether state anxiety had changed, due to treatment consumption, the score provided on Day 1 was subtracted from Day 28, providing a change score for each of the four treatments. A Tukey corrected univariate ANOVA compared these change scores and a significant effect of treatment was found; F (1, 136) = 4.42, *p* = 0.005 which revealed a significant difference between the change score of placebo and 950 mg Greek mountain tea (*p* = 0.022, *d* = 0.75) and between Ginkgo and 950 mg Greek mountain tea (*p* = 0.028, *d* = 0.77). See Figure 7 for effect of treatment on change in state anxiety.

### 4.3. Blood Pressure (BP)

No significant main or interaction effects involving treatment were observed. The data for these scales is provided in Table S1.

### 4.4. Near-Infrared Spectroscopy (NIRS)

For ease of interpretation, Epochs 1–2 refer to CBF during the 5 min rest period prior to completing the post-dose cognitive tasks (i.e., 2 × 2.5 min data points) and Epochs 3–6, 7–10 and 11–14 the first, second, and third post-dose repetitions of the cognitive demand battery, respectively. Each battery repetition comprises four epochs with the first of these representing CBF during the serial 3 subtraction task (Epochs 3, 7 and 11), the second the serial 7 subtraction task (Epochs 4, 8 and 12) and the final two the RVIP task (i.e., the 2 × 2.5 min data points at Epochs 5–6, 9–10 and 13–14).

#### 4.4.1. Oxygen Saturation (Ox%)

**Figure 8:**
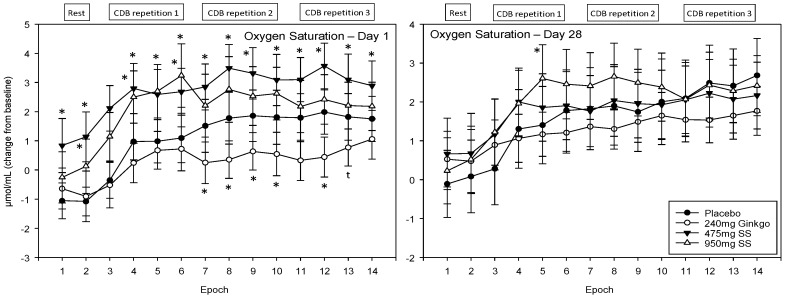
Acute and chronic effects of treatment on oxygen saturation. Mean (±SEM) oxygen saturation concentrations during 5 min of rest (averaged into 2 × 2.5 min epochs) and 30 min (three repetitions) of the cognitive demand battery (CDB). Each 10 min CDB comprises 1 × 2 min epoch of serial 3 and 1 × 2 min epoch of serial 7 subtractions and 2 × 2.5 min epochs of Rapid Visual Information Processing (RVIP). (**left**) Oxygen saturation changes on Day 1; and (**right**) oxygen saturation changes following 28 days of supplementation with 475 mg *Sideritis scardica* (SS), 950 mg SS, active control (240 mg *Ginkgo biloba*) or placebo control. Data on both days were recorded during a ~150–240 min post-dose period and converted from the pre-dose baseline on Day 1. * = *p* < 0.05.

A significant main effect of treatment was observed F (1, 1589) = 29.01, *p* ≤ 0.001 and reference to the post-hoc planned comparisons reveals that this effect was limited to acute effects (within Day 1) only. Here, Ginkgo was associated with lower saturation during the latter portion of the recording period (Epochs 7–10 and 12; *p* < 0.05, Epoch 11 *p* < 0.01 and Epoch 13 evinced a trend towards significance *p* ≤ 0.08). Both doses of Greek mountain tea evinced significantly greater saturation with the lower, 475 mg dose, producing the most pronounced effects (Epochs 1, 4, 5, 6, 8 and 12 were significant at the *p* < 0.01 level and 7, 9–11 and 13–14 at the *p* < 0.05 level for the 475 mg dose, and Epochs 1 (*p* < 0.05) 3–6 (*p* < 0.01) and 8 (trending; *p* ≤ 0.08) were significantly higher than placebo for the 950 mg dose of Greek mountain tea) See Figure 8 for acute and chronic effects of each treatment on oxygen saturation and Table S1 for complete oxygen saturation values for all treatments, on both days, alongside *p*-values and effect sizes at each epoch comparison.

#### 4.4.2. Total Haemoglobin (THb)

**Figure 9:**
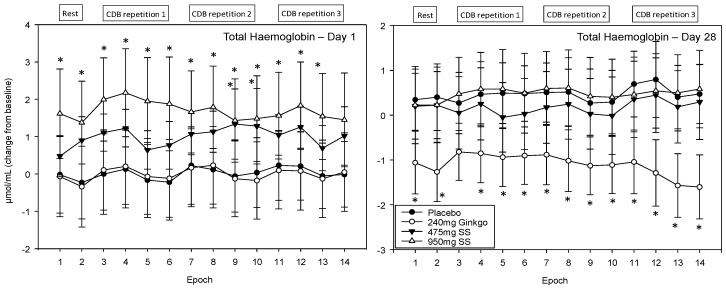
Acute and chronic effects of treatment on total haemoglobin concentrations. Mean (±SEM) total haemoglobin concentrations during 5 min of rest (averaged into 2 × 2.5 min epochs) and 30 min (three repetitions) of the cognitive demand battery (CDB). Each 10 min CDB comprises 1 × 2 min epoch of serial 3 and 1 × 2 min epoch of serial 7 subtractions and 2 × 2.5 min epochs of Rapid Visual Information Processing (RVIP). (**left**) Total haemoglobin changes on Day 1; and (**right**) total haemoglobin changes following 28 days of supplementation with 475 mg *Sideritis scardica* (SS), 950 mg SS, active control (240 mg *Ginkgo biloba*) or placebo control. Data on both days were recorded during a ~150–240 min post-dose period and converted from the pre-dose baseline on Day 1. * = *p* < 0.05.

A significant main effect of treatment was observed F (1, 1589) = 28.03, *p* ≤ 0.001 and reference to the post-hoc planned comparisons on Day 1 reveals that, for 950 mg Greek mountain tea, all 14 epochs were significantly different compared to placebo with Epochs 1–6, 8 and 12–13 at the *p* < 0.01 level and Epochs 7, 9–11 and 14 at the *p* < 0.05 level. For the 475 mg dose of Greek mountain tea, Epochs 9 and 10 were significant at the *p*< 0.05 level and Epochs 2–4 and 12 were trending towards significance (*p* ≤ 0.08). During Day 28 the only treatment to evince effects significant from placebo was Ginkgo with significantly lower levels of total haemoglobin at all 14 epochs (1 and 4–10 at the *p* < 0.05 level, 2 and 11–14 at the *p* < 0.01 level and 3 trending). See Figure 9 for acute and chronic effects of each treatment on total haemoglobin levels and Table S1 for complete total haemoglobin concentrations for all treatments, on both days, alongside *p* values and effect sizes at each epoch comparison.

#### 4.4.3. Oxygenated Haemoglobin (HbO)

**Figure 10:**
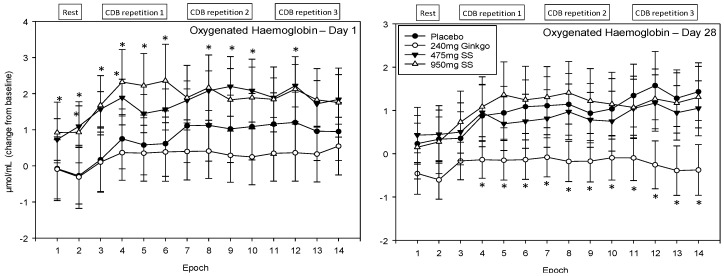
Acute and chronic effects of treatment on oxygenated haemoglobin concentrations. Mean (±SEM) oxygenated haemoglobin concentrations during 5 min of rest (averaged into 2 × 2.5 min epochs) and 30 min (three repetitions) of the cognitive demand battery (CDB). Each 10 min CDB comprises 1 × 2 min epoch of serial 3 and 1 × 2 min epoch of serial 7 subtractions and 2 × 2.5 min epochs of Rapid Visual Information Processing (RVIP). (**left**) Oxygenated haemoglobin changes on Day 1; and (**right**) oxygenated haemoglobin changes following 28 days of supplementation with 475 mg *Sideritis scardica* (SS), 950 mg SS, active control (240 mg *Ginkgo biloba*) or placebo control. Data on both days were recorded during a ~150–240 min post-dose period and converted from the pre-dose baseline on Day 1. * = *p* < 0.05.

A significant main effect of treatment was observed F (1, 1589) = 29.0, *p* ≤ 0.001 and reference to the post-hoc planned comparisons reveals a very similar pattern of effects as those outlined for total haemoglobin above. Here, again, only the two doses of Greek mountain tea evinced effects on Day 1, compared to placebo, where levels of oxygenated haemoglobin were significantly higher following Greek mountain tea. For the 475 mg dose, Epochs 2 and 3 were significant at the *p* < 0.01 level, Epochs 4, 9, 10 and 12 at the *p* < 0.05 level and 5, 6, 8 and 14 trending towards significance. For 950 mg, Epochs 3–6 were significant at the *p* < 0.01 level, 1, 2 and 8 at the *p* < 0.05 level and 12 and 13 were trending towards significance. During visit 2, Ginkgo was again associated with significantly lower levels of oxygenated haemoglobin (as total haemoglobin described above) compared to placebo and here Epochs 4–7, 9 and 10 were significant at the *p* < 0.05 level, Epochs 8 and 11–14 at the *p* < 0.01 level and Epoch 2 was trending towards significance. See Figure 10 for acute and chronic effects of each treatment on total haemoglobin levels and Table S1 for complete total haemoglobin concentrations for all treatments, on both days, alongside p values and effect sizes at each epoch comparison.

#### 4.4.4. Deoxygenated Haemoglobin (Hb)

**Figure 11:**
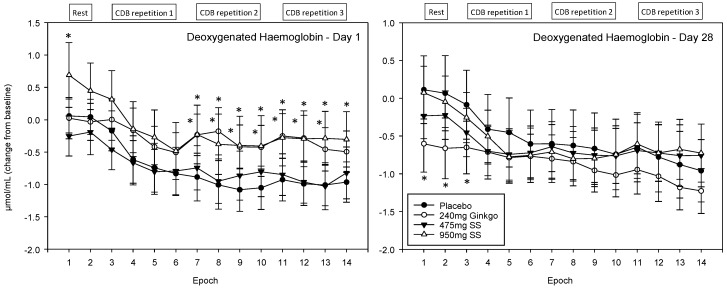
Acute and chronic effects of treatment on deoxygenated haemoglobin concentrations. Mean (±SEM) deoxygenated haemoglobin concentrations during 5 min of rest (averaged into 2 ×2.5 min epochs) and 30 min (three repetitions) of the cognitive demand battery (CDB). Each 10 min CDB comprises 1 × 2 min epoch of serial 3 and 1 × 2 min epoch of serial 7 subtractions and 2 × 2.5 min epochs of Rapid Visual Information Processing (RVIP). (**left**) Deoxygenated haemoglobin changes on Day 1; and (**right**) deoxygenated haemoglobin changes following 28 days of supplementation with 475 mg *Sideritis scardica* (SS), 950 mg SS, active control (240 mg *Ginkgo biloba*) or placebo control. Data on both days were recorded during a ~150–240 min post-dose period and converted from the pre-dose baseline on Day 1. * = *p* < 0.05.

A significant main effect of treatment was observed F (1, 1589) = 4.91, *p* = 0.027 and reference to post-hoc planned comparisons reveals that, on Day 1, these effects were seen for Ginkgo and the 475 mg dose of Greek mountain tea only and, here, both treatments evinced significantly higher levels than placebo. For Ginkgo, Epochs 7, 10 and 13 were significant at the *p* < 0.05 level, Epochs 8, 9, 11 and 12 at the *p* < 0.01 level and Epochs 4 and 14 were trending towards significance. The 475 mg dose of Greek mountain tea evinced significant effects at the *p* < 0.05 level for Epochs 1, 7, 8, 10, 11 and 14, at the *p* < 0.01 level for Epochs 9, 12 and 13 and Epochs 3–5 were trending towards significance. During visit 2, there were some small effects for Ginkgo only, at the start of the recording period, where levels were significantly lower than placebo at Epochs 1, 2 (*p* < 0.01) and 3 (*p* < 0.05). See Figure 11 for acute and chronic effects of each treatment on deoxygenated haemoglobin levels and Table S1 for complete deoxygenated haemoglobin concentrations for all treatments, on both days, alongside p values and effect sizes at each epoch comparison.

## 5. Discussion

The current study investigated whether two doses of *Sideritis scardica* (Greek mountain tea) were capable of improving aspects of cognitive function and mood in a group of healthy, older adults, and whether this coincided with modulation of CBF parameters, after acute administration and then following 28 days of consumption. The cognitive and mood results revealed that, relative to the placebo control, the higher, 950 mg dose of Greek mountain tea evinced significantly fewer false alarms on the RVIP task on Day 28 and significantly reduced state anxiety following 28 days of consumption. Compared to the active control, *Ginkgo biloba*, this higher dose of Greek mountain tea also reduced state anxiety and attenuated a reduction in accuracy on the picture recognition task, on Day 1 and Day 28. Both doses of Greek mountain tea trended towards significantly faster speed of attention on both days, relative to Ginkgo. 

In terms of CBF modulation, both doses of Greek mountain tea, relative to placebo, increased oxygenated haemoglobin (HbO) and oxygen saturation (Ox%) in the prefrontal cortex during completion of cognitively demanding tasks on Day 1. The higher dose also evinced greater levels of total (THb) and deoxygenated haemoglobin (Hb) on Day 1 but no additional effects were seen on CBF on Day 28 following either dose of Greek mountain tea. *Ginkgo biloba* led to lower levels of Ox% and higher levels of Hb on Day 1 and lower levels of both HbO and THb on Day 28.

For clarity, relative to the inactive, placebo control, it was the higher dose of Greek mountain tea only which benefited cognition and mood. On the former, effects were restricted to reducing erroneous responses on a task requiring rapid processing of information in order to detect strings of numbers (RVIP). That the ability to correctly detect these strings was not also improved suggests that 950 mg Greek mountain tea was acting to inhibit responses based on incorrect processing rather than augmenting the ability to detect the information per se. This effect was restricted to Day 28, in the absence of a main effect of day, suggesting that this was still the product of acute consumption within Day 28 itself rather than an accumulation of 28 days consumption. It is not clear why an acute effect was not also seen on Day 1, but reference to the effects on mood may shed some light here.

The 950 mg dose of Greek mountain tea evinced a significant reduction in state anxiety on Day 28 (relative to Day 1, where no effects on mood were observed), relative to placebo, with an ~3 score reduction on the STAI and a large effect size of *d* = 0.75. These effects are consistent with the berry polyphenol literature and effects which our own lab has observed recently, both with combinations of berry polyphenols (in the form of purple grape juice and berry extracts) and when isolating single phenolic acids. With regards the former, purple grape juice was found to increase ratings of calm in young, healthy humans [35] and, in a separate trial, a (green) coffee berry extract (rich in chlorogenic acid) reduced mental fatigue, inertia, confusion, bewilderment and overall negative mood, alongside increasing vigour, activity and alertness [15]. In terms of mechanisms, recent data reveal that monoamine oxidase B (MAO-B) inhibition can occur 60 min after ingestion of a berry polyphenol extract [36] and the ensuing prevention of monoamine neurotransmitter recycling may underpin the positive mood effects observed in the polyphenol literature and current trial.

Turning attention now to the active control, 240 mg *Ginkgo biloba*, the higher dose of Greek mountain tea also attenuated reduced accuracy on the picture recognition task, on both days, and both doses of Greek mountain tea trended (*p* = 0.08) towards significantly faster speed of attention, relative to Ginkgo, on both days as well. The former effect of 950 mg Greek mountain tea emerges from a general trend for all other treatments to evince reduced accuracy (relative to baseline on Day 1) at the post-dose repetition time-points on both days (both repetitions on Day 1 and the second repetition only on Day 28). Relative to this, picture recognition accuracy is higher at the abovementioned time-points, following the higher dose of Greek mountain tea. No significant main effect of day was observed in this analysis and so the consumption of 950 mg acutely on Day 1 and Day 28 underpins these effects. On Day 1, these effects coincide with significant modulations of all four CBF outcomes as assessed by NIRS. Here, consistently higher overall CBF (represented by THb), demand for oxygen (represented by HbO) and use of this oxygen (represented by Hb), as well as some small effects on Ox%, was observed following 950 mg Greek mountain tea, compared to baseline on Day 1, and relative to levels observed for placebo. This supports the ability of phenolic acids to interact with NO-induced vasodilation [11,12] and to influence blood flow parameters [14], including in the brain [15].

The CBF modulation of this polyphenol-rich Greek mountain tea is in line with effects seen after other combinations (e.g., cocoa flavanols) and single polyphenols, where NO-induced vasodilation is believed to be the underpinning mechanism. The closest example here is to those effects observed with the stilbene polyphenol resveratrol, where multiple studies by our own lab have observed robust acute effects (45–90 min post-dose) [21,37] in the absence of chronic (28 days later) effects [20]. No cognitive effects were observed in any of these trials, demonstrating the difficulty in matching the CBF mechanism to cognitive outcomes. This difficulty is seen with other polyphenol combinations, e.g., cocoa flavanols, where promising cognitive improvements are seen [24], especially in older participants [25,26], but disappear when CBF measures are attempted alongside [38]. This could suggest that the reduced power in trials utilizing neuroimaging is hindering the detection of cognitive effects and/or that this mechanism is not a robust facilitator of cognitive function—or not in all age groups. In line with this, Desideri et al. [25] linked quicker performance on a psychomotor task in a group of cognitively intact elderly participants to improved insulin resistance following eight-week consumption of 993 mg cocoa flavanols daily. Berry polyphenols (333 mg/daily/6 weeks) have also been linked to improved insulin sensitivity in non-diabetic obese 40–70 year olds [39] and, given the age-related decline in insulin sensitivity and its correlation with cognitive decline [40], this appears a more likely but underexplored mechanism of cognitive enhancement. Given the commonality between the cohort ages and chronic time-frame in the above trials and the current study, improved insulin sensitivity could be underpinning the effects seen on Day 28, thus overshadowing the CBF effects seen on Day 1, and certainly requires further investigation in future trials with this age group.

As an interesting aside, it should be noted that the effects of the active control, *Ginkgo biloba*, on CBF are counter to the received wisdom that this terpene is capable of increasing blood flow parameters. Many papers reporting cognitive effects of Ginkgo, at least in part, attribute these effects to potential enhancement of CBF [41] and this is likely based on old animal data, where blood flow effects have been found (e.g., [42]), and some tentative effects in humans (e.g., ocular blood flow effects in elderly glaucoma patients [28] and in elderly patients with cerebral insufficiency [29]). However, these effects were in relatively small, niche samples and a review by Kleijnen and Knipschild [43] found that, whilst positive effects of Ginkgo in cerebral insufficiency were consistent throughout the literature, this summary was based on serious flaws such as those mentioned above. Further, when considering the results of the only (to the best of current knowledge) trial which has attempted to measure both cognitive and CBF effects of Ginkgo, we see that, whilst tentative effects on the former were seen, this was in the absence of any corresponding effects on blood flow changes; as measured by functional magnetic resonance imaging (fMRI) [44]. In the current trial, we observed significant acute reductions in oxygen saturation and significant chronic reductions in total and oxygenated haemoglobin suggesting reduced CBF demand and provision of oxygen, by Ginkgo, respectively. However, increased use of the oxygenated blood that was present was increased on Day 1 (as indicated by higher deoxygenated haemoglobin levels). That this coincides with reduced picture recognition accuracy and slower speed of attention, on both Day 1 and Day 28, for Ginkgo, suggests that these disruptions to CBF are evincing poorer cognitive function. However, as this intervention was not the focus of this trial, and the period of intervention was much shorter than that typically utilized with Ginkgo (with more positive effects seen over these longer-term trials), future trials would need to delve deeper to investigate whether this was a robust effect or whether it was the product of an older sample, inconsistency in active components within the Ginkgo product used here compared to those previously and/or some other methodological variable/s.

Following on from this, the observation that both doses of Greek mountain tea trended (*p* = 0.08) towards significantly faster speed of attention, relative to Ginkgo, should arguably be treated with more caution than the other cognitive outcomes. This effect manifests as significantly faster performance on attention tasks (averaging numeric working memory, choice reaction time and RVIP) at both post-dose repetitions on Day 1 and the second post-dose repetition on Day 28. However, Figure 6 demonstrates that, rather than Greek mountain tea increasing the speed of attention per se, *Ginkgo biloba* appears to be slowing performance. Several studies conducted by our own lab have provided mixed results here. For example, an equivalent dose of 240 mg *Ginkgo biloba* initially evinced significant increases in speed of attention at 2.5 h post-dose [41], an effect which was not replicated in a follow up study supplementing 360 mg, despite cognitive effects seen on other domains with this dose [45]. Finally, consumption of 120 mg *Ginkgo biloba* evinced negative effects, i.e., as in the current study, a reduction in speed, on the speed of attention factor, despite positive effects on cognition elsewhere with this dose [46].

Based on the above alone, a bell-shaped curve emerges where the lower dose (120 mg) slows attentional speed, the higher dose (360 mg) exerts no effect and the mid-dose (240 mg) improves speed of attention. Why this same dose had the counter effect in the current study is not clear but a direct comparison is complicated by the fact that the samples in all of the above trials were young (18–35 years) relative to the older (mean age 60 years) cohort here. Further, as mentioned above, consistency across Ginkgo trials is hindered by a lack of consistency in the herbal extracts utilized, where the presence of bilobalide and ginkgolides, as well as other key phenolic ingredients, is not always guaranteed [47]. Interpretation of this effect should also be treated with caution as the day × treatment interaction effect that these comparisons were predicated on was only marginally trending towards significance. Nevertheless, the clear pattern of effects and the fact that the effect sizes for the significant comparisons ranged from medium to large (i.e., *d* = 0.59 to 0.74) suggests that interpreting this trend is valid and that, similar to other terpenes, e.g., *Melissa officinalis* (lemon balm) [48], Ginkgo may exert sedative-like effects at particular doses/depending on chemical make-up/in particular cohorts.

In summary, the most salient effect reported here was the significant reduction in anxiety evinced by 28 days consumption of 950 mg *Sideritis scardica* (Greek mountain tea). RVIP false alarms were also reduced by this dose on Day 28. This was the product of acute consumption of treatment on Day 28 and could be underpinned by the aforementioned mood effect seen following 28 days of consumption. Both 950 and 475 mg Greek mountain tea significantly increased CBF parameters on Day 1 but not Day 28, supporting the role of this as a potential acute mechanism which is not required to bolster cognition long-term. The active control, *Ginkgo biloba*, attested to the sensitivity of the methodology by demonstrating acute and chronic modulation of CBF; although this was counter to the received wisdom that Ginkgo enhances CBF (in short-term application). Ginkgo also provided a sensitive comparison to Greek mountain tea with regards cognitive performance. Here, in comparison, the latter led to better picture recognition, speed of attention and improved state anxiety and, taken together, the data support the role of un-caffeinated Greek mountain tea in potentially supporting everyday information processing, memory, attention and mood. One key limitation of this trial is that potential sex differences were not factored in to the design of the trial and so information regarding stage of menstruation and menopause, and medications pertinent to both, is not available; nor were sufficient numbers/ratios of participants recruited to allow for sub-group analyses related to these factors. Future trials should certainly include sex differences in trial designs and, based on the findings here, interrogate further the mood effects of the higher dose in facilitating quicker information processing to more quickly discard incorrect/erroneous content. Further, whilst the lower dose was less impactful in this paradigm, it certainly still warrants further attention. Observing the above effects of *Sideritis scardica* after only ~150 min (CBF effects on Day 1), ~9 and 310 min (cognitive effects on both days, relative to both controls) and quite a stark anxiolytic effect following only 28 days consumption, suggests that this intervention is capable of quite rapid acute effects; future trials might also consider extending this trial period to ascertain if these benefits persist or even strengthen over time.

## Figures and Tables

**Table 1:** ANOVA and Bonferroni corrected post-hoc *t*-test results summary for significant cognitive outcomes. Table summarises the day (D) and day × treatment (D*T) interaction effects from the chronic ANOVAs (effects compared across treatments at pre-dose on Day 28) and repetition (R) and repetition × treatment (R*T) interaction effects from the acute ANOVAs (effects within Day 1 and Day 28) as well as the Bonferroni corrected post-hoc *t*-tests and effect sizes from the latter for the three significant cognitive outcomes. Here, G = *Ginkgo biloba* and P = placebo versus (V) the *Sideritis scardica* (475 mg and 950 mg) treatments.

Measure	ANOVAs						Post-Hoc Comparisons		
	1. Pure Chronic ANOVA			2. Acute Effects within Day 1 and Day 28 ANOVA					
	Effect	F	P	Effect	F	P	Treatment Comparisons	P	Effect Size
Picture Recognition Accuracy (% correct)	D	2.60	0.11	R	11.66	<0.001 *	G V 950 mg Rep 1 G V 950 mg Rep 2 G V 950 mg Rep 5	0.02 0.03 0.01	0.68 0.61 0.79
	D*T	0.94	0.42	R*T	1.84	0.04 *			
Rapid Visual Information Processing False Alarms (number)	D	2.41	0.12	R	3.01	0.03 *	P V 950 mg Rep 4	0.02	0.66
	D*T	2.16	0.10	R*T	2.08	0.03 *			
Speed of Attention (milliseconds)	D	31.10	<0.001 *	R	2.47	0.05 ^t^	G V 475 mg Rep 1 G V 950 mg Rep 1 G V 475 mg Rep 2 G V 950 mg Rep 2 G V 475 mg Rep 5 G V 950 mg Rep 5	0.01 0.05 ^t^ 0.09 ^t^ 0.02 0.02 0.01	0.66 0.54 0.51 0.63 0.59 0.74
	D*T	0.51	0.68	R*T	1.65	0.09 ^t^			

* *p* < 0.05; t = trend.

## Supplementary Materials

The following are available online at http://www.mdpi.com/2072-6643/10/8/955/s1, Table S1: Means and standard deviations (alongside in italics) of cognitive task performance during day 1 and day 28. Baseline and pre-dose data is raw whilst post-dose data is change from baseline.
